# Investigating a cluster of pediatric oncology invasive fungal infections–Lessons learned

**DOI:** 10.1017/ash.2022.167

**Published:** 2022-05-16

**Authors:** Angelette Terk, Jennifer Ormsby, Paula Conrad, Catherine Svensson, David Barry, David Davis, Ana Vaughan Malloy, Tom Sandora

## Abstract

**Background:** In spring 2021, the infection prevention and control department at a pediatric academic medical center identified 3 oncology patients with concern for invasive *Rhizopus* spp infections. An in-depth investigation was conducted, but a common source of the fungus was not identified. In August 2021, an additional oncology patient with concern for invasive *Rhizopus* spp was identified, resulting in an extended investigation for possible sources of fungus. **Methods:** A multidisciplinary work group was assembled. The CDC Targeted Environmental Investigation Checklist for Outbreaks of Invasive Infections Caused by Environmental Fungi was used as a framework for conducting the investigation. Stakeholders were engaged throughout the process, including the hematology–oncology service, hospital leadership, environmental services, patient safety and quality, and facilities and engineering. The investigation included hospital incident command system (HICS) activation; visual inspection of patient rooms and common spaces; heating, ventilation, and air conditioning (HVAC) review; environmental sampling (surfaces, linen, and air); chart review; and process mapping. **Results:** By early October 2021, 2 environmental samples grew isolates (each at 1 CFU/m^3^) of the same species of *Rhizopus* as one of the affected patients. One sample was from a patient room, and the other from an outdoor garden space. No source of indoor amplification of *Rhizopus* was identified. The investigation revealed several opportunities for improvement: annual room maintenance schedules, use of gardens and outdoor spaces by at-risk patients, linen storage, construction and/or infection control risk assessment (ICRA) processes, and appliances used by families (eg, washing machines and refrigerators). Work streams were established to address each of these areas. **Conclusions:** No definite source was identified for the 4 invasive *Rhizopus* spp infections. This extensive investigation highlighted multiple opportunities for improvement; the changes implemented may prevent future invasive fungal infections in high-risk pediatric patients.

**Funding:** None

**Disclosures:** None

